# An Alternative Rescue Procedure for Neuroform Atlas® Withdrawal During Deployment: A Report of Two Cases

**DOI:** 10.7759/cureus.71755

**Published:** 2024-10-18

**Authors:** Ryuzaburo Kanazawa, Takanori Uchida, Tetsuhiro Higashida, Noboru Kuniyoshi

**Affiliations:** 1 Neurosurgery, Tokyo General Hospital, Tokyo, JPN; 2 Neurosurgery, Nagareyama Central Hospital, Chiba, JPN; 3 Internal Medicine, Otakanomori Station Clinic, Chiba, JPN; 4 General Internal Medicine, Nagareyama Central Hospital, Chiba, JPN

**Keywords:** endovascular technique, neuroform atlas, troubleshooting, unruptured aneurysm, withdrawal during deployment

## Abstract

Neuroform Atlas^®^ (NFA; Stryker Neurovascular, Fremont, CA, USA) is a useful and safe device for the treatment of broad-necked and unruptured cerebral aneurysms. Rarely does a proximal shift of both the stent and delivery catheter occur during deployment, and it can be complex to treat. We present two cases in which an NFA that had migrated proximally during deployment was successfully retrieved.

In Case 1, a left internal carotid artery (ICA) and posterior communicating artery (Pcom A) bifurcation aneurysm of 12.8 mm in maximum diameter was treated by the stent-assisted technique. An NFA stent (4 ´ 21 mm) was selected for use after placement of three coils. During deployment of the stent from the left Pcom to the ICA, withdrawal of the system in the proximal direction resulted in part of the stent falling into the aneurysm. The half-released stent was retrieved carefully because we considered that complete deployment of the stent would result in inadequate treatment and fatal consequences in the long term. After Case 1, an experiment was conducted to determine whether it was safe to retrieve the NFA into the parent catheter. In Case 2, we experienced the same situation during the procedure of an anterior communicating artery aneurysm. Based on the experience of Case 1, we were able to perform stent retrieval in Case 2 without hesitation.

The sudden withdrawal of an NFA stent can occur during the placement procedure. If surgeons encounter proximal migration during NFA deployment, retrieval of the NFA may be an option.

## Introduction

The Neuroform Atlas® (NFA; Stryker Neurovascular, Fremont, CA, USA) stent has a low-profile structure that enables it to be introduced into distal vessels, enabling deployment through a 0.0165-inch inner diameter microcatheter [1-10]. Its application for stent salvage during coil embolization has been reported [8], and as good clinical results have been demonstrated in stent-assisted coil embolization [1-4,6,7,9], we consider this device to be effective and safe based on literature reports, our experience, and the fact that treatment options have expanded. Despite the widespread use of the NFA stent, a small but significant number of adverse events, including stent migration, have been reported, with potential risks of severe complications [5,10,11]. Although most of these clinical events did not affect clinical outcomes, the operator must handle such situations appropriately.

Here, we describe a case of microcatheter withdrawal inside an aneurysm, which occurred during half-deployment of an NFA stent (4 ´ 21 mm), after which the stent was retrieved (Case 1). An experiment was conducted to confirm whether or not stent retrieval is possible and safe in this situation. A subsequent stent retrieval in a patient is then described (Case 2). Informed consent was obtained from each patient for the descriptions in this study and the use of their radiological images. The two procedures and the experiment were performed by the same surgeon.

The purpose of this study is to propose an option for treating problems that occur during NFA deployment, especially when the NFA dislodges proximally.

## Case presentation

Case 1

**Figure 1:**
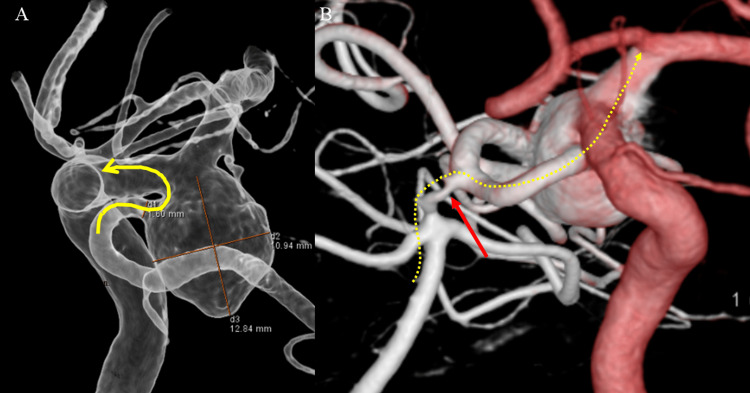
Preoperative plan (A) Stent placement is from the Pcom A to the proximal ICA (yellow curved arrow). (B) In Plan 2, stent placement is from the terminal ICA to the Pcom A after advancing the microcatheter through the narrow P1 (yellow dotted arrow). In Plan 1, the stent placement route has a hairpin curve for smooth deployment, whereas Plan 2 has a risk of ischemia during the procedure due to the narrow proximal part of the PCA (P1) (red arrow). After careful consideration, Plan 1 was selected as the first choice, with Plan 2 as the backup plan. Pcom A: posterior communicating artery, ICA: internal carotid artery, PCA: posterior cerebral artery

A 78-year-old female with a left internal carotid artery (ICA) and posterior communicating artery (Pcom A) junction aneurysm was admitted to our hospital. Mild hypertension was seen in her past medical history. No habits of tobacco or alcohol were demonstrated. The parent vessel diameter was 3.4 mm, the neck was 5.3 mm, and the dome-to-neck ratio was 2.1. The Pcom A diameter was 1.6 mm, which was too small for NFA placement, but we considered that there was no effective treatment method other than placing a stent in the Pcom A. We also determined that the Pcom was relatively thick, and the risk of thrombotic occlusion associated with stent placement was not high. After receiving full informed consent, we decided on the treatment plan. In determining the treatment strategy prior to the procedure, we considered two stent placement routes: from the Pcom A to the ICA (Plan 1) (Figure 1A) and from the ICA terminal to the Pcom A after introducing a delivery catheter from the left proximal portion (P1) of the posterior cerebral artery (PCA) (Plan 2) (Figure 1B). In Plan 1, the route for stent placement had a hairpin curve for smooth deployment, whereas in Plan 2, there was a risk of ischemic complications due to the narrow P1. After careful consideration, Plan 1 was selected. Because stent placement prior to coil insertion was not considered safe, we planned to insert some coils in advance to support the microcatheter for stent deployment. Finally, Plan 1 was considered the first choice, followed by Plan 2 as the backup plan. For both plans, it was considered important to place the support catheters as distally as possible to ensure optimal maneuverability of the microcatheter.

**Figure 2:**
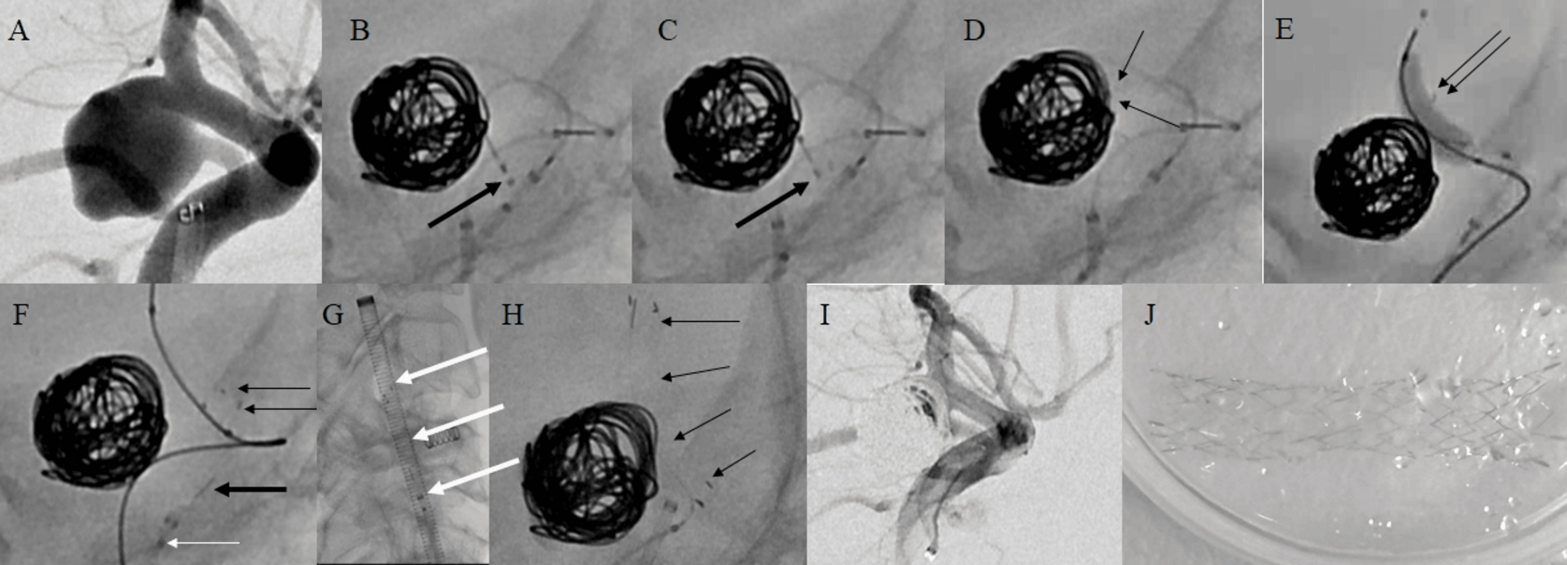
Fluoroscopic images during treatment of a left ICA and Pcom A aneurysm in Case 1 (A) A fetal-type Pcom A is identified, originating near the neck of the aneurysm. Release of the stent is seen initially in the Pcom A. (B) The thick black arrow indicates the distal marker on the stent. (C) The stent is unfolded at the distal site (thick black arrow). (D) Withdrawal of the stent is observed, with the end of the stent entering the aneurysm and changing the formation of the coil (thin black arrows). (E) Stent retrieval is attempted using a balloon catheter to avoid displacing the coils. The thin arrows indicate the distal markers on the stent. (F) The fluoro-image of the balloon deflated after the stent was retrieved outside the aneurysm. The coil mass and stent are completely separated. The stent is retrieved into the guiding catheter without interference from the coil or balloon catheter that accompanies stent retrieval afterward. Thin black arrows, unfolded part of the stent; white arrow, marker on the microcatheter tip; black bold arrow, the tip of the delivery wire. (G) The stent is successfully stored inside the guiding catheter (thick white arrows). (H) Another stent is then placed from the terminal ICA to the Pcom A (thin black arrows). (I) The final image shows the successful completion of the procedure. There is no apparent arterial dissection due to retrieval of the stent. (J) Retrieved stent in Case 1. There is no remarkable damage to the device. ICA: internal carotid artery, Pcom A: posterior communicating artery

The treatment procedure is shown in Figure 2. Dual support catheters (TACTICS 3.2/3.4 Fr, 120 cm; Technocrat Corporation, Aichi, Japan) were introduced to the cavernous portion of the ICA through a 7 Fr guiding sheath (7 Fr Shuttle Sheath, 80 cm; Cook Medical, Bloomington, IN) positioned at the left ICA origin (Figure 2A), followed by insertion of a stent delivery catheter (Excelsior XT-17 1.7/2.4 Fr, 150 cm; Stryker) to the Pcom A and a coil delivery catheter (Excelsior 1018 2.0/2.6 Fr, 150 cm, Stryker) into the aneurysm. After inserting three coils (two Axium Prime Frame Complex coils, 10 mm ´ 30 cm, Medtronic Neurovascular, Irvine, CA) and one Target 360 XXL coil, 8 mm ´ 20 cm, Stryker), a stent (NFA 4 mm ´ 21 mm) was deployed (Figure 2B-2C). However, just after half of its length had unfolded in the Pcom A, the stent fell inside the aneurysm (Figure 2D). After deliberation, we conducted stent retrieval due to the risk that complete deployment of the stent would result in incomplete obliteration and adversely affect the patient’s long-term outcome. After withdrawing the Excelsior 1018 and TACTICS microcatheters, a balloon catheter (Scepter C 4 mm ´ 10 mm, Terumo Corporation, Aliso Viejo, CA) was introduced to occlude the neck of the aneurysm to avoid dislodging the coils (Figure 2E). The stent was then carefully retrieved with the delivery wire locked to the microcatheter under balloon inflation (Figure 2F), followed by successful retrieval of the system (Figure 2G). A 6 Fr guiding catheter (6 Fr Launcher 90 cm, Medtronic Neurovascular) was introduced via the left femoral artery to the right vertebral artery and a TACTICS microcatheter was navigated proximal to the terminal end of the basilar artery. The same stent release procedure was performed successfully via an XT-17 stent delivery catheter navigated from the left PCA and Pcom A to the left terminal end of the ICA (Figure 2H). Residual coil embolization was carried out through a microcatheter (Excelsior SL-10 90 degree, Stryker) from the ICA with the trans-cell technique. No adverse events, such as artery dissection, occurred intraoperatively (Figure 2I). Postoperative diffusion-weighted imaging showed only two small, asymptomatic high signals, and the patient was discharged one week after the procedure with no neurological deficit. No clinical adverse events or recurrence of the aneurysm have occurred during follow-up. The retrieved stent was distorted but not damaged (Figure 2J). The postoperative course was uneventful, and the patient has been free of recurrence for 5 years since the operation.

Experiment

**Figure 3:**
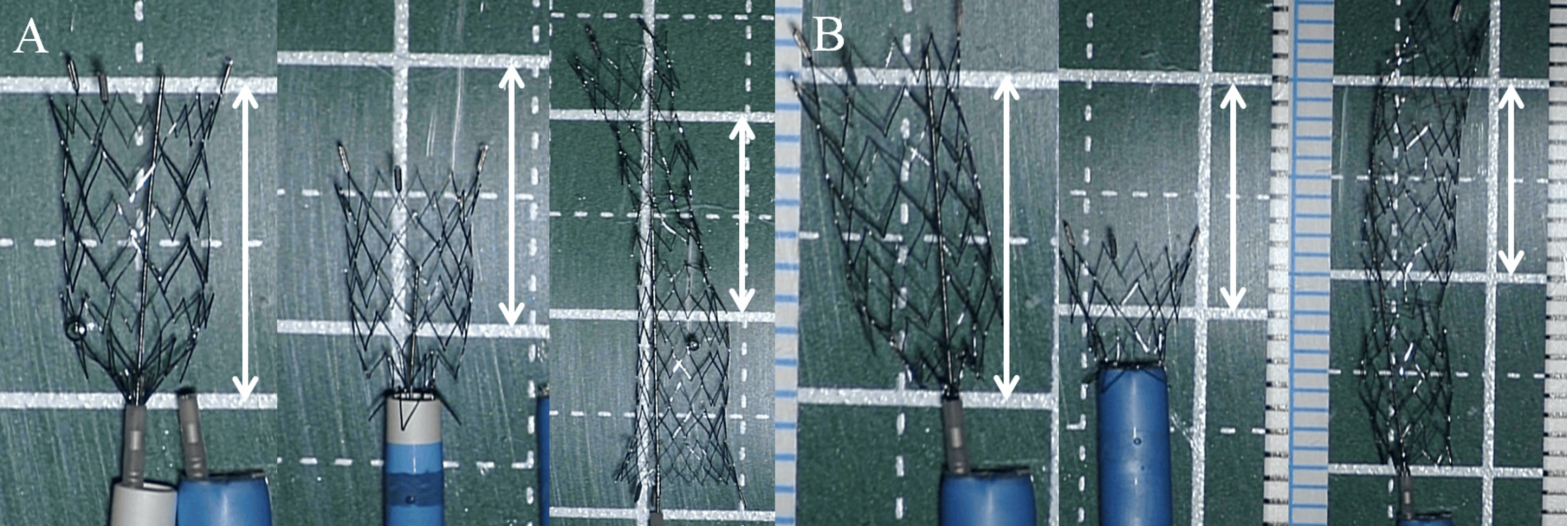
Photographs showing the condition of stents at each stage of the retrieval experiment, using (A) a 6 Fr Launcher and (B) a 7 Fr Shuttle Sheath In each experiment, an NFA stent (4 ´ 21 mm) is unfolded to the same length as in a procedure, approximately 10 mm (left images), and then drawn into the catheter manually. The stent is stored in the catheter without any difficulty (middle images). Images of the entire length of the stent (right images) show damage to the stent, but no fragmentation is seen. All white arrows indicate a length of 10 mm. NFA: Neuroform Atlas®

To confirm the safety and reproducibility of retrieval of the stent inside the guiding catheter, we conducted an experiment with the same system used in the procedure to treat Case 1. From the analysis of images on the Picture Archiving and Communication System, we measured the length of the released stent, which was approximately 10 mm. We assessed the behavior of the NFA 4´ 21 mm stent during and after retrieval with an XT-17 microcatheter in two setups: with a 6 Fr Launcher (Figure 3A) and with a 7 Fr Shuttle Sheath guiding catheter (Figure 3B). The NFA stent was released to approximately 10 mm in length, as in the procedure for Case 1 (Figure 3A left, Figure 3B left). In both setups, the stent was retrieved safely from the guiding catheter (Figure 3A middle, Figure 3B middle) without breakage (Figure 3A right, Figure 3B right). The results of this experiment indicated that the Atlas stent could be retrieved uneventfully inside these guiding catheters. When retrieving a stent, three issues may arise: whether there is damage to the blood vessel wall at the deployment site, whether the deployed stent can be retrieved into the parent catheter, and whether the stent will fall off due to friction with the blood vessel wall and during retrieval inside the catheter when the entire system is pulled. This experiment suggested that the latter two issues are possible.

Case 2

**Figure 4:**
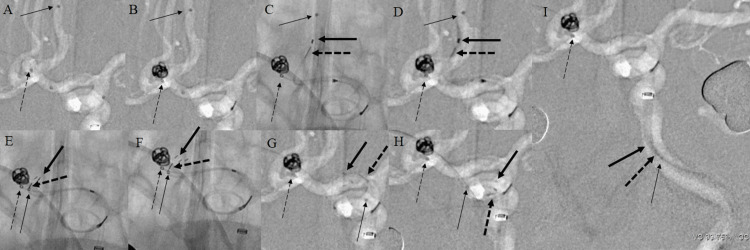
Intraoperative findings in Case 2 (A) After the microcatheter for embolization (GREACH) and the microcatheter for the stent delivery (XT-17) are introduced. (B) After the first coil was placed, the procedure was continued without detaching it. (C, D) Navigation of the NFA close to the tip of the XT-17. (C: the fluoro image, D: the roadmap image) (E) Proximal dislodgement immediately after stent deployment. (F) The image at the start of stent retrieval. The tip of the stent is partially opened. (G, H) The image shows the stent retrieval. No change in position or shape of the coil frame or the microcatheter for embolization. (I) The image of the stent after it was retrieved into the intermediate catheter. No abnormalities were found in the coil embolization system. Thin black arrow: the tip of XT-17. Thin-dotted black arrow: the tip of GREACH. Thick black arrow: the tip of the stent. Thick dotted black arrow: the tip of the stent delivery wire. NFA: Neuroform Atlas®

A 79-year-old female with an anterior communicating artery aneurysm was admitted to our hospital after radiographic follow-up magnetic resonance imaging examinations showed enlargement of the aneurysm. The patient had been prescribed statin and did not have other lifestyle-related diseases. No habits of tobacco or alcohol were demonstrated. Elective embolization was performed using a 6 Fr Shuttle Sheath (Cook) guiding catheter, a 6 Fr Sofia Select (Terumo) support catheter, a GREATH 1.7/2.4 Fr microcatheter (Tokai Medical, Aichi, Japan) for coil insertion, and an XT-17 (Stryker) stent delivery catheter (Figure 4A-4B). A 3 ´ 21 mm NFA stent was selected for use (Figure 4C-4D). When the stent was released in the distal anterior cerebral artery, withdrawal occurred (Figure 4E). If the stent had been deployed completely, the proximal end would have been released into the terminal ICA. As we considered that this situation could result in inadequate treatment of the aneurysm, we retrieved the stent carefully according to our earlier experience with Case 1 (Figure 4F-4I). The alternative double-catheter technique was employed, and the procedure was completed uneventfully. No obvious damage was observed at the tip of the stent. The postoperative course was uneventful, and the patient has been free of recurrence for three years since the operation.

## Discussion

The current report suggests one possible option when the NFA becomes dislodged before being placed at the desired site and suggests that the NFA may be retrieved without damaging the arterial wall if it is not released from the microcatheter. In Case 1, half of the stent had detached but was successfully retrieved, and in Case 2, it was possible to retrieve it from a smaller blood vessel.

The NFA is a low-profile and highly trackable stent [1-10]. The open-cell structure is simple to deploy, and the landing position is predictable [7]. In addition, the proximal end has a closed-cell structure to facilitate the use of microcatheters to secure the true lumen [3,6,8]. Several studies have reported adequate aneurysmal obliteration of 88.5-96.6% following NFA stent procedures, with thromboembolic complications reported in 3.3-14.8%, most of which were transient or asymptomatic [1-4,6,7,9].

However, several studies have reported a small number of technical failures, including the following: The first is the proximal withdrawal of the stent and microcatheter, similar to our experience, during the release or introduction of the stent [1,4-6,9]. This phenomenon has been reported in 1/55 (1.8%) NFA procedures performed by Baek et al. [1], in 1/31 (3.2%) by Kim et al. [4], in 2/123 (1.6%) by Kwon and Chung [6], in 1/27 (3.7%) by Ten et al. [9], in 1/113 (0.9%) by Caragliano et al. [3], and in 2/65 (3.1%) performed at our hospital. The second includes the more distal position of the proximal end of the stent than expected, resulting in migration of the end into the aneurysm [7,10] or movement of the stent inside the aneurysm by friction during the introduction of the microcatheter in the trans-cell technique [2,10]. More distal deployment has been reported in 1/36 procedures performed by Tomio et al. (2.8%) [10] and in 1/30 (3.3%) by Quintana et al. [7]. In contrast, stent migration to the proximal end during coil delivery introduction by microcatheter has been reported in 1/128 (0.8%) by Burkhardt et al. [2] and in 1/36 (2.8%) by Tomio et al. [10]. Third, the stent can stretch during deployment for no apparent reason [5]. Ko and Shin reported unexpected stretching in stent lengths of up to 40 mm (NFA 4 mm ´ 21 mm) [5]. In addition, Yanagawa et al. reported entanglement of the stent and the microwire [11], with proximal slippage of the stent during microwire retrieval, which was followed by successful additional stent placement with no adverse events such as vessel dissection. These phenomena might be interpreted as the fragility of this device due to its more sophisticated and delicate structure compared with the original Neuroform EZ (Stryker) [10]. Fourth, the microwire can damage the stent during the trans-cell technique [11]. Withdrawal and proximal migration of the system are the most common of these adverse events. Both continuation of the procedure after stent deployment [7,10] and stent retrieval [9] have been reported after the occurrence of withdrawal, although the latter study reported few details. The authors stated that the partially deployed stents were removed by locking the stent to the microcatheter and retracting both the microcatheter and the partially deployed stent simultaneously [9]. These findings suggest that stent retrieval might be feasible if it is determined that stent retrieval could be performed safely without interfering with other devices.

In the case that the SL-10 stent delivery microcatheter cannot adequately support the system in a tortuous vessel [9], Baek et al. recommended that the XT-17 be used [1], whereas we used an XT-17 stent delivery catheter in both of the present cases. As the abrupt withdrawal of the stent and microcatheter proximally during deployment has been reported to occur in 0.9-3.2% of procedures [3,4,6,9], the operator should be aware of the possibility of this event prior to NFA placement. In relation to deployment, more distal than predicted, during deployment the length of the stent is mainly around the aneurysmal neck rather than in the daughter or parent vessel. Therefore, it is important to pay attention to the initial position at release. Careful manipulation of the microcatheter or wire inside the lumen of the stent is essential to prevent damage from the stent [7,10,11].

If withdrawal of the system occurs with half-release of the stent, it is possible to retrieve the system by locking the delivery wire and delivery microcatheter [9], as was performed in the present patients and in our experiment. After successful retrieval, unnecessary add-on anti-platelet treatment can be avoided, along with restoration of the preoperative situation. To the best of our knowledge, there are no reports of significant vessel injury by NFA stent migration, possibly due to the mild radial force of the stent. In our opinion, retrieval is a viable option in troubleshooting, but until now it was unknown how much of the unfolded length could be retrieved. According to our experience and findings of the present experiment, 10 mm is the approximate maximum length of the unfolded stent that can be retrieved for a 4 ´ 21 mm NFA, but the retrievable length is unknown for greater than half release.

This study has limitations. These include the fact that it was based on only two cases, and there is no evidence as to whether stent retrieval is possible without complications such as vascular dissection. Although various troubles have been reported during NFA placement, fortunately, there have been no reports of vascular damage that can occur at the same time, so it could be considered that stent retrieval may be an option depending on the situation.

## Conclusions

The NFA stent is an effective and safe device for the treatment of cerebral aneurysms in various locations. It is necessary to decide how to deal with adverse events that may occur during NFA deployment after carefully considering whether the surgery can be completed at an acceptable level and the long-term prognosis after treatment. If the NFA is not completely detached, it is also important to decide how to deal with the situation after considering whether the stent can be safely retrieved.
